# Characterization of sperm and implications for male fertility in the last of the Rhynchocephalians

**DOI:** 10.1093/conphys/coad071

**Published:** 2023-08-30

**Authors:** Sarah K Lamar, Nicola J Nelson, Diane K Ormsby

**Affiliations:** School of Biological Sciences, Victoria University of Wellington, Level 2 Te Toki a Rata Building, Wellington 6012, New Zealand; Centre for Biodiversity and Restoration Ecology, Victoria University of Wellington, Level 2 Te Toki a Rata Building, Wellington 6012, New Zealand; School of Biological Sciences, Victoria University of Wellington, Level 2 Te Toki a Rata Building, Wellington 6012, New Zealand; Centre for Biodiversity and Restoration Ecology, Victoria University of Wellington, Level 2 Te Toki a Rata Building, New Zealand; School of Biological Sciences, Victoria University of Wellington, Level 2 Te Toki a Rata Building, Wellington 6012, New Zealand

**Keywords:** Curvilinear velocity, morphology, reproductive conservation, reptiles, sperm, sperm motility, sperm viability

## Abstract

Managing a species of conservation concern can be best achieved when there is information on the reproductive physiology of both sexes available; however, many species lack this critical, baseline information. One such species, the tuatara (*Sphenodon punctatus*), is the last surviving member of one of the four reptile orders (Rhynchocephalia) and is the only reptile known to lack a male intromittent organ. Culturally and evolutionarily significant, the conservation of this species is a global priority for the maintenance of biodiversity. In light of this, we characterized the morphology, viability and swim speed of mature tuatara sperm for the first time. We found that tuatara sperm are filiform and bear the remarkably conserved three-part sperm structure seen across the animal kingdom. Tuatara sperm are long (mean total length 166 μm), with an approximate head:midpiece:tail ratio of 15:1:17. While tuatara sperm are capable of high levels of within-mating viability (94.53%), the mean viability across all samples was 58.80%. Finally, tuatara sperm had a mean curvilinear velocity swim speed (μ × s − 1) of 82.28. At the population level, there were no differences in viability or mean swim speed between sperm collected from a male’s first mating of a season and repeat matings; however, the maximum sperm swim speed increased in observed repeated matings relative to first matings. Interestingly, faster sperm samples had shorter midpieces, but had greater viability and longer head and tail sections. This work expands our understanding of male reproductive characteristics and their variation to a new order, provides wild references for the assessment of captive individuals, lays the groundwork for potential assisted reproductive techniques and highlights variation in male reproductive potential as an important factor for consideration in future conservation programs for this unique species.

## Teaser

We identified considerable variation in male fertility in wild tuatara, an evolutionarily unique reptile. Our findings show that tuatara sperm morphology is relatively conserved but sperm viability and swim speed vary significantly among matings. Thus, we strongly suggest that variation in male fertility be considered during future conservation actions.

## Introduction

Sexual selection, historically viewed as competition between members of the mate-limited sex for reproductive opportunities (Darwin, 1871), can be better understood as competition over access to the limited gamete (Lüpold *et al.,* 2020; Parker, 2020). This definition allows for the inclusion of variation in both mating and fertilization success and is particularly important in systems with multiple mating, sperm storage or post-copulatory sexual selection (Firman *et al.,* 2017; Lüpold *et al.,* 2020). Cornerstone to this theory is the understanding that the sex with the more numerous gametes, typically males, should have a selective advantage when they produce either higher quality or larger quantities of that gamete. In males, investing energy to increase sperm production can be accomplished by growing larger testes or, often, by producing large quantities of smaller sperm (Parker, 1982; Lüpold *et al.,* 2020). This theoretical reduction in sperm size is thought to be the underpinning behind anisogamy (Parker, 1978, 1982), and thus knowledge of the structure and function of gametes from across the tree of life is at the core of sexual evolutionary theory and understanding the persistence of species.

The general three-part structure of sperm is remarkably conserved across vertebrate lineages, with each section playing a distinct role in the cell’s function (Horta *et al.,* 2018). Sperm cells consist of a head containing genetic material packaged as chromosomes within the sperm’s nucleus, a midpiece housing mitochondria that provide the sperm with the energy necessary to move, and a tail that propels the cell towards the unfertilized egg (Horta *et al.,* 2018; Kahrl *et al.,* 2022). Because each section serves a unique function within the sperm cell, these sections may respond differently to varying selection pressures (Reinhardt *et al.,* 2015). Subsequently, variation in the length and shape of each sperm section can be observed across taxa (Horta *et al.,* 2018), between modes of fertilization within a taxa (Kahrl *et al.,* 2021, 2022) and in response to the intensity of selection pressures such as cryptic female choice and inter-male sperm competition (hereafter ‘sperm competition’) (Firman *et al.,* 2017; Lüpold *et al.,* 2020). These structural changes can also influence the speed at which sperm move; for example, the sperm midpiece houses the mitochondria that produce ATP, the energy source for sperm movement (Lüpold and Pitnick, 2018; Lara *et al.,* 2020). However, whether sperm diversification is occurring via selection acting directly on the cell or by selecting for optimal male and female microenvironments that elicit a plastic response in sperm is not yet fully resolved (Reinhardt *et al.,* 2015). In general, theory suggests that higher levels of sperm competition may result in an increase in both the overall length of sperm and the length of each section (Briskie *et al.,* 1997; Fitzpatrick *et al.,* 2009); this covariance could be the result of genetic or other functional and mechanistic constraints. However, recent research has found that sperm sections can display distinct rates of phenotypic diversification (Immler *et al.,* 2012; Rowe *et al.,* 2015; Kahrl *et al.,* 2019; Fitzpatrick *et al.,* 2020; Friesen *et al.,* 2020; Reuland *et al.,* 2021), suggesting that the independent evolution of sperm sections is possible (Kahrl *et al.,* 2022), though this is poorly understood in many taxa. Thus, it is important to consider variation in the length of each section independently.

Sperm viability, approximated as cell membrane integrity, and sperm motility, measured as % motile sperm and/or sperm swim speed, are perhaps the two most important sperm functional traits in determining male fertilization success (Gasparini *et al.,* 2010; Boschetto *et al.,* 2011; Fitzpatrick and Evans, 2014). There is broad support for an increase in sperm swimming speed in species with high levels of sperm competition (Lüpold *et al.,* 2009; Fitzpatrick, 2020). However, theory predicts a trade-off between sperm viability and swimming speed, though experimental support of this prediction is mixed (Malo *et al.,* 2005; Smith, 2012; Silva *et al.,* 2017), suggesting that multiple *in vivo* factors likely mediate the relationship. These factors include cryptic female choice, length of sperm storage and the period that gametes interact (Sever and Hamlett, 2002; Firman *et al.,* 2017; Fitzpatrick, 2020). Importantly, selection is unlikely to impact a single sperm trait in isolation, and thus measures of sperm morphology, viability and motility must all be considered when investigating the evolution of mating systems, particularly in taxonomically or ecologically unique species without experimental congeners.

Tuatara (*Sphenodon punctatus*) are the sole surviving members of Rhynchocephalia, a once diverse and widespread order of reptiles (Fraser, 1988; Daugherty *et al.,* 1990; Cree, 2014; Gemmell *et al.,* 2020). Endemic to Aotearoa New Zealand, natural populations of tuatara remain only on offshore satellite islands free from invasive mammalian predators (Gaze, 2001; Cree, 2014). Tuatara diverged from their closest kin, squamate reptiles, approximately 250 million years ago during the Triassic (Gemmell *et al.,* 2020). In addition to a suite of unique skeletal and dental characteristics, tuatara are the only reptiles that lack an intromittent organ (penis or hemipenis) to help deposit sperm inside the female reproductive tract and instead mate via cloacal apposition (Cree, 2014). Mating occurs at the end of the austral summer (late February–March), when males undertake elaborate mating displays called ‘stolzer Gangs’ (Gillingham *et al.,* 1995; Moore *et al.,* 2009). If a female is receptive, the male mounts the female and uses his rear limbs to rotate her pelvis and align their cloacae to deposit sperm. The male typically remains mounted on the female for a significant period (reported up to 90 minutes; Moore *et al.,* 2009), though sperm transfer is suspected to occur within the first 5–10 minutes of mounting the female (SKL unpublished data). Interestingly, ovulation occurs approximately 1–2 months after mating and female tuatara have no known organ, or oviductal crypts, for long-term sperm storage (Gabe and Saint Girons, 1964; Moore *et al.,* 2009). Despite the lack of known sperm storage organ, polyandry, polygyny and multiple paternity have all been observed in the species, as well as monandrous clutches being laid by socially polyandrous females (Moore *et al.,* 2009). Thus, tuatara sperm are likely experiencing the selective pressures of sperm storage, sperm competition and cryptic female choice.

Until recently, our only knowledge of Rhynchocephalian sperm came from a 1992 study looking at the ultrastructure of non-viable sperm excised from the testes of two euthanized tuatara (Healy and Jamieson, 1992). However, in 2021 a pilot study investigating methods of cryopreservation for tuatara sperm was carried out using seven sperm samples collected from males living on Takapourewa (Stephens Island), Cook Strait, New Zealand (Lamar *et al.,* 2021). This study found that of the seven collected sperm samples, five were motile, and one sample had the fastest (at the time of publication) curvilinear velocity of any reptile sperm (Lamar *et al.,* 2021). Additionally, sperm samples had the potential for very high viability (Lamar *et al.,* 2021). However, this study had a small sample size and focused largely on the effects of different buffers and cryopreservatives on tuatara sperm. Tuatara are a managed species through New Zealand’s Department of Conservation and are under threat from developing a male bias due to temperature dependent sex determination and ongoing anthropogenic climate change. Thus, reproductive research that may improve the selection of individuals for inclusion in conservation actions, such as translocations and captive breeding programs, is critical.

In light of these threats and the opportunity to fill a large phylogenetic gap in our understanding of reptile reproductive characteristics, we undertook a study with three primary goals. First, using samples collected across two mating seasons, we characterized Rhynchocephalian sperm morphology, viability and swim speed in a robust, systematic way for the first time. Second, to quantify the amount of intra-individual variation in tuatara sperm functional characteristics, we investigated whether tuatara sperm characteristics varied across multiple mating events involving the same male. We hypothesized that sperm quality would decline throughout the mating season due to the energetic demands associated with sperm production and mating behaviour. Finally, we tested for the presence in trade-offs between sperm morphology, viability, and swim speed. We suspected that, like birds, longer sperm would likely have faster swim speeds; however, this might come at the expense of investment in membrane formation or susceptibility to damage associated with an increase in surface area, leading to faster sperm samples having lower levels of overall viability.

## Materials and Methods

We carried out the following research under Wildlife Act Authority permit #50568-FAU and Victoria University of Wellington animal ethics committee permits #27041 and #30011.

We collected sperm from mating tuatara on Takapourewa (Stephens Island), Cook Strait, New Zealand, in two consecutive years during the austral summer. In 2021, we collected samples between 18 February and 6 March; in 2022, we collected samples from 18 February to 11 March. To collect sperm samples, we conducted visual surveys starting at approximately 16:00 and ending when activity quieted for the night, at approximately 02:00 hours. When we encountered a mating pair of tuatara, we hand captured the individuals and collected any sperm present from the pair’s cloacae using a sterile syringe (approx. volume ≤ 0.015mLs). If the pair was spotted as mating began (identified by the male mounting the female), they were allowed to mate for approximately 5–15 minutes before being disrupted to allow for sperm deposition.

We viewed sperm samples using an Eclipse E200 Nikon microscope and a 40× objective to visually assess approximate concentration; we then diluted samples in sterile phosphate-buffered saline (PBS) until sperm were able to be seen in a single layer when dropped on a glass microscope slide. Dilutions ranged from approximately 1:2 to 1:3 sperm:PBS. We then divided sperm samples into three aliquots. First, we pipetted 1.5 uL of sperm sample onto a clean microscope slide for morphology analysis. We allowed this slide to air dry before fixing it with 95% ethanol. Second, we combined 10 uL sperm sample with 10 uL Sperm VitalStain™ (Nidacon), which contains both eosin and nigrosine, and created duplicated smears for viability analysis. Finally, within 15 minutes of sample collection, we pipetted 6 uL of sample into a 20 μm, two chambered Leja® slide (LSC-20-01-02-B; Gytex Pty Ltd) for motility analysis. We recorded videos of sperm swimming at ambient temperature using a Gigabit Ethernet camera (Basler Scout ACA780-75GC) attached to an Eclipse E200 Nikon microscope with a 40× objective set to negative phase and Sperm Class Analyzer software v6.0.0.1 (SCA, Microptic). Previous work indicates that PBS buffer does not influence sperm velocity in tuatara (Lamar *et al.,* 2021).

From each tuatara we collected a suite of morphometric data before releasing individuals at their capture location, including snout-vent length (SVL) and weight. We calculated body condition index (BCI) as the ratio of log transformed SVL to log transformed weight.

**Figure 1:**
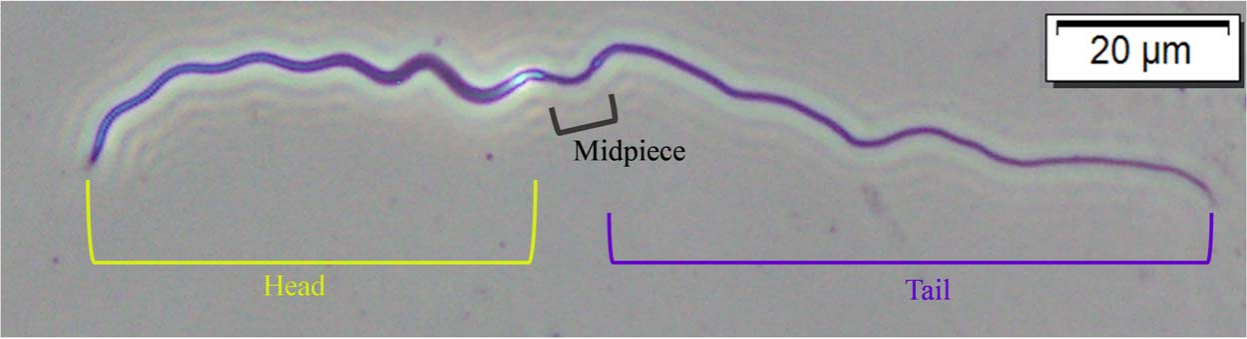
Photograph of a tuatara sperm taken at 400x magnification with major sections denoted.

Once back on the mainland, we assessed sperm morphology for all mating events for which sample quality allowed. To do this, we used Nikon Eclipse E200 microscopes with 400x phase contrast and cellSens (Olympus) software. First, two technicians measured the head, midpiece, tail and total lengths of the same 30 sperm for three mating events (total of 90 sperm with two sets of repeated measurements) (Fig. 1). We tested for technician precision using paired *t*-tests and found no significant difference between repeated technician measurements. Thus, technicians divided up the remaining mating events and read 15 sperm cells per mating. Slide quality for some mating events (*n* = 6) prevented the measurement of 15 sperm cells; for four mating events no morphology measures were collected and for two mating events fewer than 15 sperm cells were measured (10 and 6, respectively).

We sealed sperm viability slides using DPX mountant (Sigma-Aldrich) and a coverslip. Once slides were dry, two observers made duplicate counts of live and dead sperm cells for 12 randomly selected mating events (2 slides per mating event, 24 total slides) using Nikon Eclipse E200 microscopes with 40× objectives and tally counters. Observer counts did not vary by more than 13% for any slide (mean ± standard error (se) = 3.60 ± 0.76%) and duplicate slides from the same mating event did not vary from each other by more than 13% (mean ± se = 3.40 ± 1.10%). Moving forward, only one observer conducted viability counts and only one slide was read for each mating event. To assess viability, we counted 100–150 sperm cells per mating event depending on cell quantity and slide quality, with the exception of six samples. One sample was unable to be read due to poor quality and for five additional mating events we were only able to count between 59 and 92 cells due to small sample volume. We report viability as the % of live cells. Finally, we measured the curvilinear velocity (VCL) of each sperm sample using the videos of sperm swimming recorded in the field and SCA software. We manually verified that every sperm identified by CASA software was a sperm cell and not an artefact.

### Statistical analysis

We conducted all statistical analyses in R v 4.2.2 (R Core Team, 2022) and made all figures using the *ggplot2* R package (Wickham, 2016). The alpha level for all analyses was set to 0.05. First, using the *psych* and *plotrix* packages in R (Lemon, 2006; Revelle, 2017), we calculated mean and standard error (se) for the SVL and BCI of our mating male tuatara to establish population characteristics. We assessed normality of these metrics using Shapiro–Wilk tests and tested for differences in these metrics between sampling years using unpaired Welch’s *t*-tests.

To investigate the morphological structure of tuatara sperm, we report the mean, se and range for the head, midpiece, tail and total lengths of sperm from each mating event. We tested for normality of the head, midpiece, tail and total length measures using a series of Shapiro–Wilk tests; we used a series of Wilcoxon tests to test for differences in each measure between years. To quantify the amount of variation in the length of each sperm section, we calculated the coefficients of variation for these four morphological measures.

We used a Shapiro–Wilk test to assess normality of viability data and a set of Wilcoxon tests to investigate for differences in viability between years and between samples collected during first and repeat mating events by the same male. We calculated the mean, se, and range for all viability samples and used the *stats* package (R Core Team, 2022) to construct a univariate linear model for all males with multiple observed mating events. We compared sperm viability with the observed mating event number, which was treated as an ordinal value.

To assess the swim speed (curvilinear velocity, VCL; μ × s − 1) of tuatara sperm, we first calculated the number of sperm measured per sample via the SCA software. Of the 59 collected samples, we were able to measure the velocity of ≥100 sperm cells from 50 samples. The other nine samples ranged from 6–86 measured cells, reflecting low quantities of sperm collected during these mating events. Using the 50 more robust samples, we calculated between- and within- mating event variance and intraclass correlation (ICC) for the following groups of fastest sperm for each sample: 1–5, 1–10, 1–20, 1–50 and 1–100. We calculated ICC as the proportion of between-mating variance to the total phenotypic variance (between-mating + within-mating variance) (Nakagawa and Schielzeth, 2010; Lara *et al.,* 2020) and *z*-transformed all VCL values before analysis. Based on the results of this analysis, we carried out all further velocity analyses using the five fastest sperm for each mating event. Because all 59 samples had at least five measurable sperm, we report sperm velocity measures for all collected samples (*n* = 59 mating events, total of 295 sperm cells).

We tested for normality of tuatara sperm VCL values using a Shapiro–Wilk test; we used a Wilcoxon test to assess for differences in VCL between collection years and between first and repeat mating events by the same male. We calculated summary statistics (mean, se, and range) for VCL values for each mating event. Next, we fit univariate linear models for the minimum, maximum, and mean VCL for each male with multiple mating events against the mating event number.

Finally, to investigate for the presence of any trade-offs or relationships between our sperm measures, we constructed a generalized linear mixed model with mean VCL as the response variable and % viability and head, midpiece and tail lengths as predictor variables; we included year as a random effect. For each model with a Δ AICc < 6, we calculated predictor variable summary weights. We constructed models in R using the *lme4* and *MuMIn* packages (Bates *et al.,* 2015; Bartoń, 2022).

## Results

We collected 20 sperm samples from 14 tuatara in 2021 and 39 sperm samples from 24 tuatara in 2022 (total of 59 samples from 38 males). Males were observed to mate between 1 and 7 times; however, most males that were observed mating multiple times mated twice (10 out of 13). No males mated across both years. Male SVL (*t* = −0.58, df = 27.28, *P* = 0.570) and BCI (*t* = 0.50, df = 28.62, *P* = 0.620) measures were normally distributed and did not differ significantly between sample years (*P* ≥ 0.199) (Table 1), and thus were not included as a factor in analyses investigating sperm characteristics.

**Table 1:** Morphometric data from males included in this study

	Total	2021	2022
*SVL (mm)*			
Mean (± se)	258.03 (± 2.08)	256.43 (± 3.47)	258.96 (± 2.64)
Range	230.0–280.0	230.0–274.0	236.0–280.0
*BCI*			
Mean (± se)	1.17 (± 0.004)	1.17 (± 0.006)	1.17 (± 0.005)
Range	1.10–1.21	1.12–1.21	1.10–1.21

There was no difference in either measure between study years (2021, *n* = 14; 2022, *n* = 24).

There was no difference between technician measurements for head, midpiece, tail or total lengths (*P* ≥ 0.323). We were able to assess sperm morphology for 55 out of 59 mating events. We report summary statistics in Table 2; mean measures (μm) for each section were as follows: head = 76.59, midpiece = 4.56 and tail = 84.70. All sperm morphological measures were abnormally distributed (*P* ≤ 0.001), and all measures differed significantly between sample years (*P* ≤ 0.001). The coefficients of variation for sperm measures were as follows: head length = 17.14, midpiece length = 14.08, tail length = 10.10 and total length = 9.97.

**Table 2:** Summary statistics for tuatara sperm morphology, viability and curvilinear velocity (of the five fastest sperm/mating; see text for further detail) measures across all matings

Sperm measure	Total	2021	2022
*Head length (μm)*			
*n*	55	19	36
Mean (± se)	76.59 (± 0.46)	66.75 (± 0.27)	81.92 (± 0.57)
Range	49.97–146.53	53.20–89.49	49.97–146.53
*Midpiece length (μm)*			
*n*	55	19	36
Mean (± se)	4.56 (± 0.02)	4.63 (± 0.04)	4.52 (± 0.03)
Range	1.83–7.42	3.17–6.26	1.83–7.42
*Tail length (μm)*			
*n*	55	19	36
Mean (± se)	84.70 (± 0.30)	84.04 (± 0.36)	85.06 (± 0.42)
Range	19.92–134.40	58.15–122.08	19.92–134.40
*Total sperm length (μm)*			
*n*	55	19	36
Mean (± se)	166.0 (±0.58)	155.4 (± 0.47)	171.1 (± 0.75)
Range	105.00–264.9	132.4–202.3	105.0–264.9
*Viability (%)*			
*n*	58	20	38
Mean (± se)	58.80 (± 3.08)	64.44 (± 5.74)	55.83 (± 3.56)
Range	3.38–94.53	22.39–94.53	3.38–86.86
*Curvilinear velocity (μ × s − 1)*			
*n*	59	20	39
Mean (± se)	82.28 (± 2.64)	52.49 (± 2.59)	97.56 (± 3.27)
Range	0.00–218.41	0.00–102.55	0.00–218.41

There was no difference between sampling years for sperm viability; there were differences between years for all other measures.

**Figure 2:**
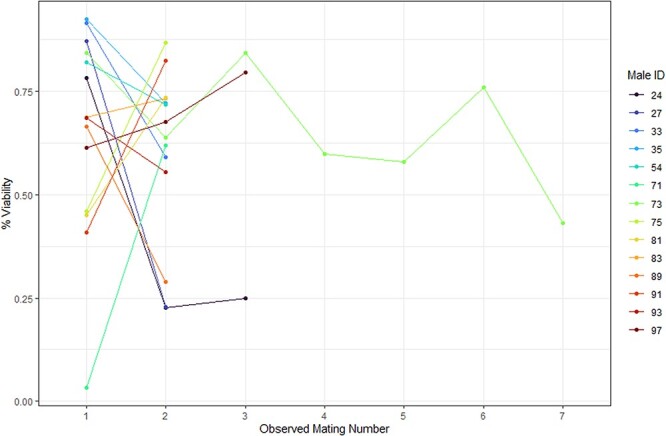
Viability (%) measures for sperm samples collected from males with observed multiple matings. No male was observed mating in both sampling years.

We were able to assess the viability of 58 out of 59 sperm samples. Viability data was abnormally distributed with a left-skew (W = 0.942, *P* = 0.008) and was not able to be brought into normality with transformation. Tuatara sperm samples collected in this study had a very large range of % viability (3.38–94.53%) (Table 2). Using Wilcoxon tests, we found no difference in % viability between years (W = 473, *P* = 0.131) or between first samples and samples collected during repeated mating events (W = 371, *P* = 0.785). The coefficient of variation for sperm % viability was 39.87. Finally, we found no relationship between the mating event number for each male and sperm sample viability (*P* = 0.595, R^2^ = 0.009) (Fig. 2).

**Figure 3:**
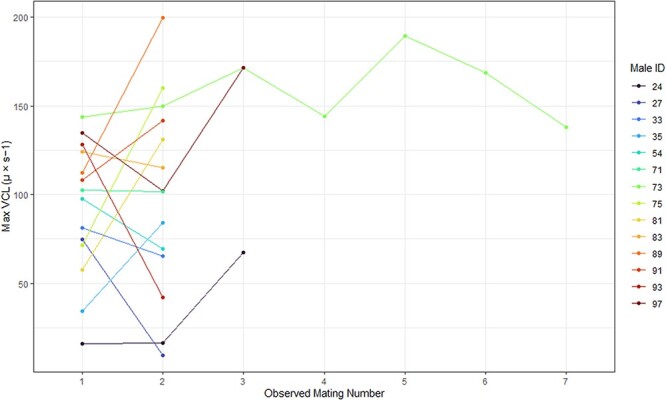
Maximum curvilinear velocity (VCL) values (μ × s − 1) for the five fastest sperm/mating for males with observed multiple matings. No male was observed mating in both sampling years.

The between-mating variance of tuatara sperm swim speed (VCL) was highest (0.819), and within-mating variance was lowest (0.200), when analysing the 1–5 fastest sperm in each sample. As the number of sperm cells analysed for each mating increased, the between-mating variance decreased steadily (1–100 sperm: 0.564) and the within-mating variance increased (1–100 sperm: 0.431) (Table 3). Considering only the five fastest sperm from each mating event, the VCL of tuatara sperm in this study was abnormally distributed with a right skew (W = 0.98, *P* = 0.002) and differed significantly between collection years (W = 3588, *P* < 0.001). However, sample size varied significantly between years and the range of VCL values observed in 2022 encompassed the range measured in 2021. There was no difference between tuatara sperm VCLs from first and repeated mating events from the same male (*t* = −1.65, df = 195.49, *P* = 0.100). Like the trend observed in % viability, there was a very large range of VCL values in this study (Table 2). We found no relationship between the mating event number for each male with multiple mating events and either the mean VCL (*P* = 0.128, R^2^ = 0.069) or minimum VCL (*P* = 0.418, R^2^ = 0.020). However, there was a relationship between the maximum VCL value and the mating event number (*P* = 0.013, R^2^ = 0.174), with an increase in maximum VCL value being observed in later mating events relative to the first mating event for each male (Fig. 3). Coefficients of variation for each sperm swim speed metric were as follows: minimum VCL (55.25), maximum VCL (49.12) and mean VCL (51.72).

**Table 3:** Variance values calculated for different subsets of fastest sperm per sample

*n*	Between-mating variance	Within-mating variance	ICC
1–5	0.819	0.200	0.803
1–10	0.748	0.261	0.748
1–20	0.713	0.291	0.713
1–50	0.634	0.336	0.634
1–00	0.564	0.431	0.564

**Figure 4:**
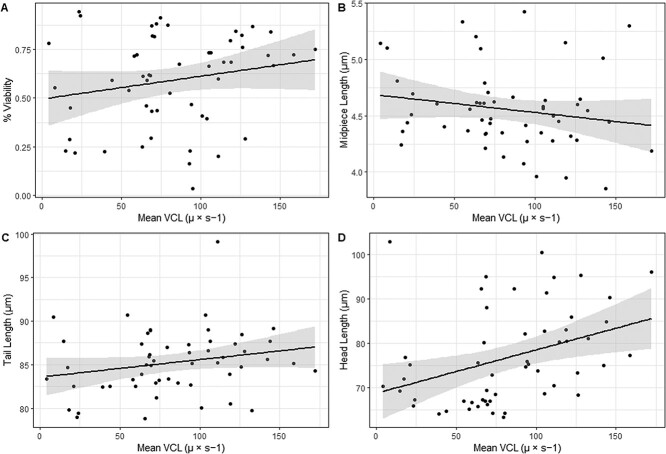
Scatterplots displaying the relationship between mean curvilinear velocity (VCL) values (μ × s − 1) and four other sperm metrics: (A) % viability, (B) midpiece length (μm), (C) tail length (μm) and (D) head length (μm). Lines display the linear relationship between variables and shaded regions denote standard error.

Five models had Δ AICc < 6 (Table 4). Viability and midpiece length were the most significant predictors of sperm swim speed (Table 5), though tail and head lengths also displayed relationships. Viability, tail length and head length were all positively related to sperm swim speed, while midpiece length was negatively related to sperm swim speed (Fig. 4).

**Table 4:** Model formulas and fixed effect model weights comparing tuatara sperm VCL swim speed (μ × s − 1) with viability (%) morphological measures (μm); we report results for all models with a Δ AICc < 6

Response variable	Model formula	Intercept	AICc	Δ AICc	Fixed effect model weight
Mean VCL (μ × s − 1)					
	Midpiece length + tail length + viability + (1 | year)	−26.34	521.3	0.00	0.419
	Midpiece length + viability + (1 | year)	86.9	522.1	1.09	0.243
	Head length + midpiece length + tail length + viability + (1 | year)	−24.96	523.1	1.83	0.168
	Head length + midpiece length + viability + (1 | year)	80.68	524.2	2.89	0.099
	Tail length + viability + (1 | year)	−66.06	526.6	5.28	0.3

**Table 5:** Sum of weights and for predictor variables found in all models with a Δ AICc < 6

Variable	Sum of weights	*n* (out of 6)
Viability	1	5
Midpiece length	0.97	4
Tail length	0.64	3
Head length	0.28	2

## Discussion

We present the first robust characterization of Rhynchocephalian sperm, using samples collected from the sole surviving member of the order, the tuatara (*S. punctatus*). While superficially similar to lizards, tuatara are the only reptiles to lack an intromittent organ and mate via cloacal apposition (Cree, 2014). Male and female tuatara remain engaged in copulation for extended periods of time (observed up to one hour), postulated as a mechanism to ensure sperm transfer in light of the lack of a penis or hemipenes in the species (Lamar *et al.,* 2021). One historic study examined sperm excised from the testes of euthanized tuatara (Healy and Jamieson, 1992); however, many animal species, including squamates in general, complete sperm maturation in the vas deferens (Jones, 1998). Though they diverged on their evolutionary pathways approximately 250 million years ago, squamates are the closest living relatives to Rhynchocephalians. Thus, sperm excised from the testes of Rhynchocephalians, and not yet having travelled through the vas deferens, are likely immature and may not accurately reflect the characteristics of sperm involved in egg fertilization in this species. The results of this study greatly improve our understanding of tuatara reproduction and provide key reference metrics for male gametes. These references can be used to help assess the fertility of animals held in captivity and/or being considered for inclusion in conservation actions.

Tuatara sperm display the same filiform, three-part structure conserved in many vertebrates (Healy and Jamieson, 1992; Lamar *et al.,* 2021). Sperm ultrastructure in reptiles is best described in Crocodilia, and all crocodilian species that have had their sperm characterized displayed a filiform structure similar to other reptiles, birds and monotremes (Horta *et al.,* 2018; Johnston *et al.,* 2020). The length of different tuatara sperm sections displayed moderate variability, with head length varying the most. While our relatively broad ranges for each section length encompassed the mean values provided in the historical study of excised testicular sperm in a small number of tuatara (Healy and Jamieson, 1992), our mean values were significantly different for each section length, particularly the head and midpieces. For example, the mean midpiece length calculated in our study was 4.56 μm vs the previously reported range of 7–8 μm (Healy and Jamieson, 1992). We attribute these differences to the historical study’s small sample size and use of immature testicular sperm.

Though the overall filiform shape is conserved, there is considerable variation in sperm length among reptile species. For example, our mean total length was 166.0 μm, and the following total length measures (μm) have been found in other species: 50–55 in the painted turtle (*Chrysemus picta*) (Hess *et al.,* 1991), 88.96 in the estuarian crocodile (*Crocodylus proosus*) (Fitri *et al.,* 2020), 90.6 in the Argentine boa (*Boa constrictor occidentalis*) (Tourmente *et al.,* 2011) and 159.3 in the Wagler’s snake (*Waglerophis merremii*) (Tourmente *et al.,* 2011). The functional effects of variation in sperm total length are mixed in the literature. In cichlid fishes, longer sperm are faster and associated with higher levels of female promiscuity (Fitzpatrick *et al.,* 2009). In New World blackbirds (Lüpold *et al.,* 2009), longer midpieces are associated with faster sperm; in two families of passerines (Fringillidae and Sylviidae) longer midpieces were associated with longer flagellum, which should correlate with sperm swim speed (Immler and Birkhead, 2007). However, the expected positive relationship between sperm competition and midpiece size was not supported in the two families of passerines studied (Immler and Birkhead, 2007). There is not currently enough research on sperm morphology in reptiles, particularly midpiece length, to examine patterns in selective pressures on midpiece length and swim speed robustly. Regardless, theory predicts that sperm size should increase when it confers benefits during sperm storage (Lüpold *et al.,* 2020), which is rife in the taxa. The comparatively long length of tuatara sperm may reflect the promiscuous nature of female tuatara, which are known to mate with multiple males and display within-clutch multiple paternity, though the presence of a sperm storage organ or specialized region of the oviduct has yet to be found (Moore *et al.,* 2009; Cree, 2014). Relative to other sperm measures assessed in our study, morphology displayed relatively low variation across all samples.

Interestingly, we identified a weak negative relationship between sperm swim speed and midpiece length when controlling for the annual effect observed in our data. Both tail and head lengths were positively related with sperm swim speed, however. Mitochondria, which are housed in the sperm midpiece, are frequently laminated in reptiles, thought to be an adaptation to aid in the maintenance of sperm viability and membrane integrity during the frequent periods of long-term sperm storage in this taxon (Hess *et al.,* 1991; Horta *et al.,* 2018). In snakes, a positive association between midpiece length and the strength of sperm competition exists, though this same pattern is not observed in lizards (Friesen *et al.,* 2020). Snakes also have a significantly longer period of sperm storage on average than is found in lizards, with the Javan file snake (*Acrochordus javanicus*) holding the record for longest known period of sperm storage among all animals (7 years—Magnusson, 1979; Friesen *et al.,* 2020). Whether longer midpieces are more common in snakes with high levels of sperm competition due to conferring an advantage during extended periods of sperm storage, or because they relay another type of advantage, remains unknown. Longer head and tail lengths were associated with faster swimming tuatara sperm. While an increase in head length, in the absence of an associated increase in tail length, can increased drag and reduce sperm swim speed (Rowe *et al.,* 2015), genetic links for a positive association between sperm swim speed and sperm length have been found in some species (Mossman *et al.,* 2009).

Tuatara sperm samples collected in this study had a broad range of viability (3.38–94.53%), measured as the % of cells with intact plasma membranes. The only other study looking at tuatara sperm viability, which included six unique tuatara and had samples collected during mating, from palpated urine, and from urine after courting, showed a similar range (0–98% viable cells) (Lamar *et al.,* 2021). The mean viability score for tuatara sperm collected across two mating seasons was 58.80%, which is lower than many other studies conducted on reptiles. For example, the mean viability scores for six neotropical pit viper species ranged from 71.1–87.6% (Blank *et al.,* 2022), for the colubrid snake subspecies *Erythrolamprus poecilogyrus sublineatus* viability ranged from 67–92% with a mean of 80%, and for the critically endangered golden lancehead viper (*Bothrops insularis*) the mean of viable sperm cells in fresh samples was 84.33%. However, the Louisiana pinesnake (*Pituophis ruthveni*) had mean % viability scores similar to those found in tuatara (means of 56–88% in four randomly assigned subgroups). Thus, while there is not a broad understanding of reptile sperm viability, these metrics provide us some context for the variation clearly present in sperm viability among lepidosaurs and highlight the need to assess the viability of sperm from males being held for captive breeding programs or moved as part of translocation programs to establish new populations.

Many extrinsic and intrinsic factors can influence a species’ sperm viability, including nutrient limitation and heterozygosity. Recent meta-analyses suggest that sperm viability is fairly robust to the effects of nutrient limitation and resource restriction, though seminal fluid quality responded strongly to resource manipulation (Macartney *et al.,* 2019). However, in experiments carried out on the brown anole (*Anolis sagrei*), sperm morphological measures and sperm count reacted strongly to nutrient restriction and experimental manipulation of body condition in males (Kahrl and Cox, 2015). The other factor often affecting sperm viability is genetic makeup of the donor individual. For example, a study controlling for body weight and comparing sperm viability in an extremely inbred population of dusky gopher frog (*Lithobates sevosus*) with low genetic diversity and an outbred, genetically diverse sister species, the northern leopard frog (*Lithobates pipiens*), found significant differences in mean sperm viability (41.96 vs 85.76%, respectively) (Hinkson and Poo, 2020). Tuatara are currently distributed across the archipelago of New Zealand and are found in disparate, isolated populations that exhibit strong genetic structuring and have relatively low levels of genetic diversity (Gemmell *et al.,* 2020). Without samples from other populations of tuatara, we cannot determine if the viability scores reported in this study are typical of the entire species, or how much variation this trait may carry. Regardless, we found that tuatara sperm viability varies less than sperm velocity and is on the lower end of what has been recorded in many wild, reptile species.

Sperm motility has been studied more widely in reptiles than other sperm characteristics, but studies with robust sample sizes are rare and many studies use less than ten individuals. While there is a broad range in reported reptile curvilinear velocities, most studied species having a mean VCL around 50 μ × s − 1 (e.g. mean of 58.97 in *B. c. occidentalis* (Tourmente *et al.,* 2007); mean of 54.16 in *Caiman crocodilus fuscus* (Valverde *et al.,* 2019). The mean VCL of tuatara (82.28 μ × s − 1), calculated using 59 samples, is the largest sample size we can presently identify for a reptile study investigating sperm swim speed. The results of our study confirm those of a pilot study suggesting that tuatara sperm have very high swim speed relative to other reptiles (Lamar *et al.,* 2021). Interestingly, we found an increase in the maximum VCL of sperm as the number of observed matings for that individual increased. Importantly, we recognize that we likely missed repeat matings for many of the males in our study due to the *in situ* nature of this sample collection, and thus mating events identified as the first for that individual, or males identified as having only mated one time, are approximations.

Many factors can influence a sperm’s swim speed, such as seminal fluid makeup (Poiani, 2006), traits within the female reproductive tract, or fertilization method (Kahrl *et al.,* 2021). In an earlier study, authors posited that the fast swim speed of tuatara sperm may be an adaptation to the lack of a male copulatory organ in the species (Lamar *et al.,* 2021). Because tuatara are both phenotypically unique among their class and the only surviving members of their order, evolutionary comparison of the driving selection pressures leading to a fast sperm swim speed is difficult. We urge future work on tuatara, particularly in relation to the conflicting trends observed in changes to % viability and maximum sperm swim speed with repeated matings, be carried out. Ideally, studies on multiple tuatara populations able to be observed across entire mating seasons, with a robust number of repeat sample collections, should be carried out to investigate the relationship between the energetic costs of repeat mating and the production of viable, fast sperm.

In summary, tuatara sperm are filiform and, relative to other reptile species, long and fast. Despite having fast moving sperm, the average viability of tuatara sperm was on the low end of what has been observed in reptiles, though no direct trade-offs were observed when controlling for animal effects. Interestingly, a negative relationship was observed between midpiece length and sperm swim speed; tail length, head length and sample viability were positively associated with increased sperm swim speed. Our results will help inform the selection of male tuatara for translocations, population supplementations and captive breeding programs by highlighting broad variance in fertility, even among males fit enough to secure mating opportunities. As reptiles undergo an extinction crisis (Böhm *et al.,* 2013) research on their reproductive characteristics has never been more critical. In species with temperature dependent sex determination living in isolated, island populations, the looming threats of sex bias and reduced reproductive output from ongoing and worsening anthropogenic climate change are significant. While natural reproduction is preferable to reproductive management, the need for assisted reproductive techniques to ensure the maintenance of genetic diversity in wild populations may arise in the near future. Thus, this work expanding our knowledge of gamete characteristics and the factors that may be influencing them into a new order, Rhynchocephalia, is timely.

## Data Availability

Data is available at 10.6084/m9.figshare.23938260
